# Supplementary material for: Molecular interactions between Hel2 and RNA supporting ribosome-associated quality control
Source: Nat Commun. 2019 Feb 4;10:563. doi: 10.1038/s41467-019-08382-z (PMC6362110; doi:10.1038/s41467-019-08382-z)
Supplement: Supplementary file 1 — Supplementary Information [file 41467_2019_8382_MOESM1_ESM.pdf]

Supplementary Information

for

Molecular interactions between Hel2 and RNA supporting ribosome-associated quality control

Marie-Luise Winz, Lauri Peil, Tomasz W. Turowski, Juri Rappsilber and David Tollervey

Contents

Supplementary Figure 1	p. 2
Supplementary Figure 2	p. 3
Supplementary Figure 3	p. 5
Supplementary Figure 4	p. 6
Supplementary Figure 5	p. 7
Supplementary Figure 6	p. 8
Supplementary Figure 7	p. 9
Supplementary Figure 8	p. 10
Supplementary Figure 9	p. 11
Supplementary Figure 10	p. 12
Supplementary Figure 11	p. 13
Supplementary Figure 12	p. 15
Supplementary Figure 13	p. 16
Supplementary Figure 14	p. 17
Supplementary Figure 15	p. 18
Supplementary Figure 16	p. 19
Supplementary Table 1	p. 20
Supplementary Table 2	p. 20
Supplementary Table 3	p. 20
Supplementary Table 4	p. 21
Supplementary Table 5	p. 22
Supplementary Table 6	p. 23
Supplementary Table 7	p. 23
Supplementary Table 8	p. 24
Supplementary Table 9	p. 26
Supplementary References	p.30

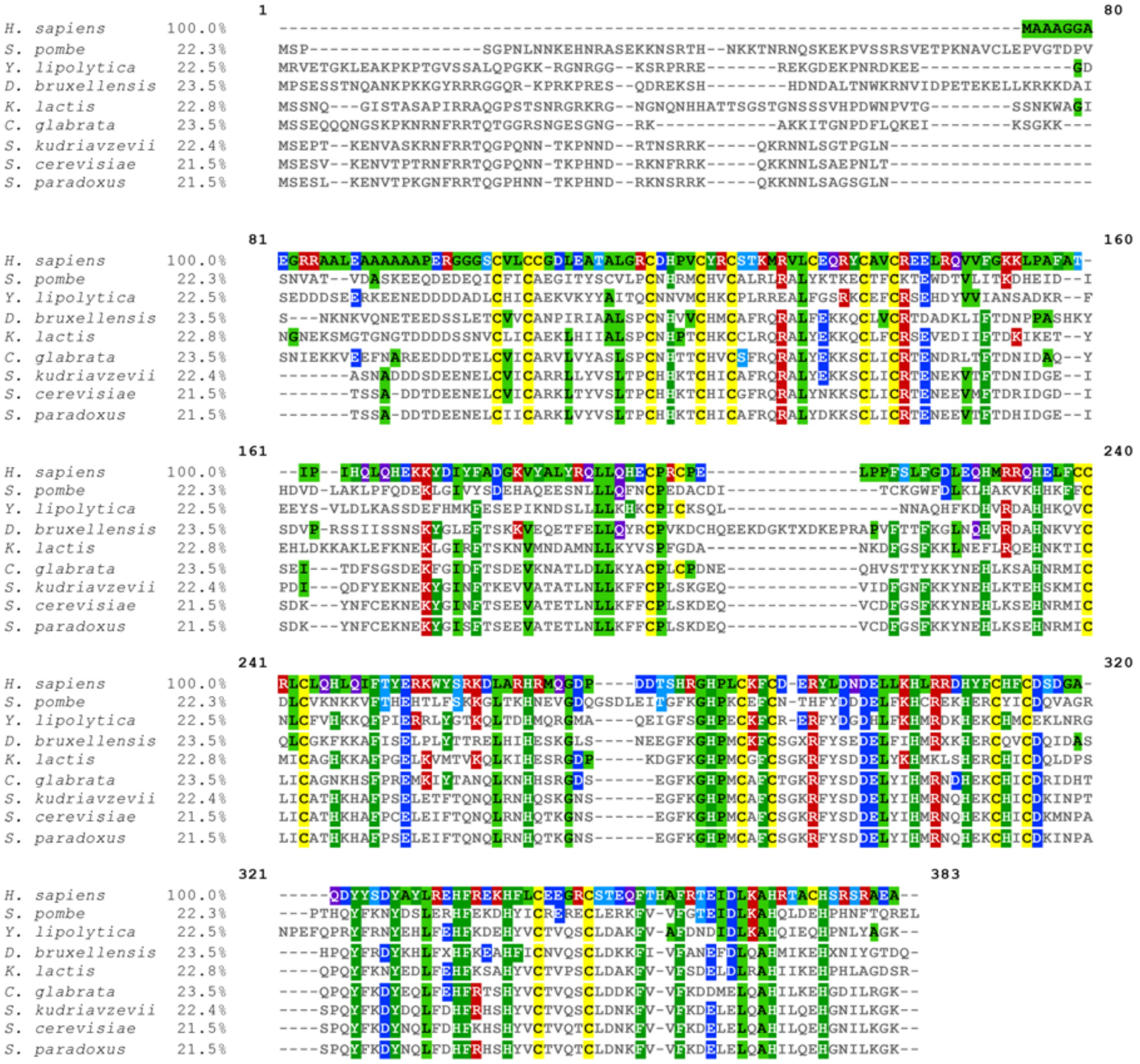

Supplementary Figure 1. Multiple sequence alignment of Hel2 and hZNF598 N-termini. Alignment was generated with Clustal Omega and visualized in MView. Amino acids are coloured by property and identity to the human sequence. Percentages next to species names designate identity to the human sequence.

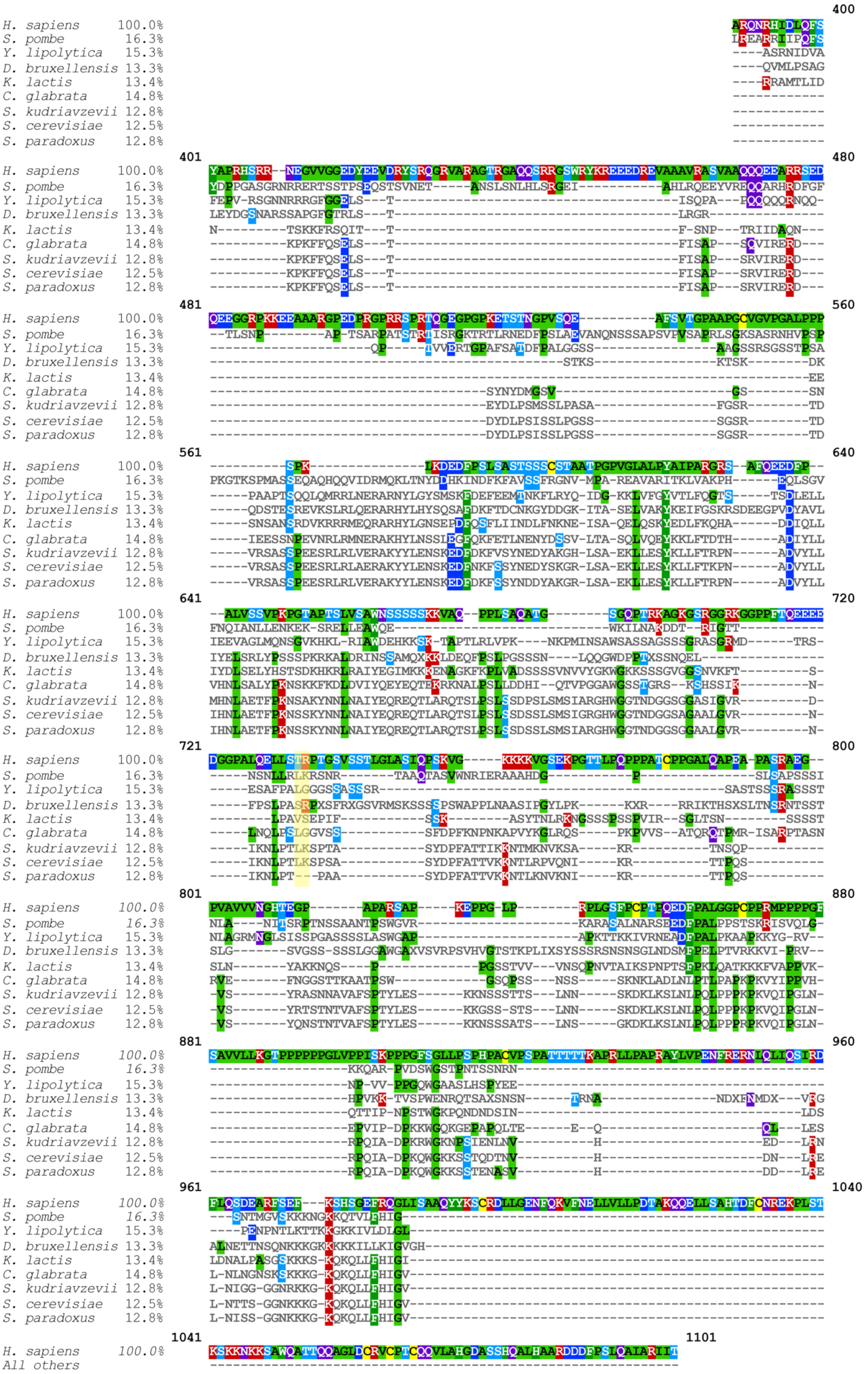
H. sapiens 100.0%
S. pombe 16.3%
Y. lipolytica 15.3%
D. bruxellensis 13.3%
K. lactis 13.4%
C. glabrata 14.8%
S. kudriavzevii 12.8%
S. cerevisiae 12.5%
S. paradoxus 12.8%
All others

Supplementary Figure 2. Multiple sequence alignment of Hel2 and hZNF598 C-termini, taken from full protein alignment (N-terminus removed after alignment). Alignment was generated with Clustal Omega and visualized in MView. Amino acids are coloured by property and identity to the human sequence. Percentages next to species names designate identity to the human sequence.

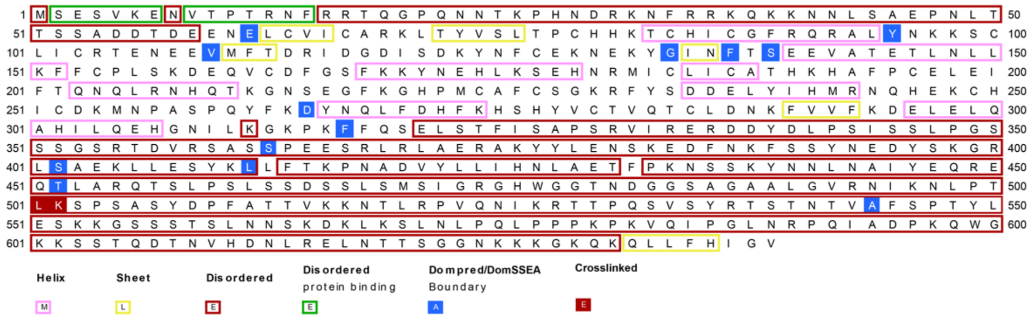

Supplementary Figure 3. Hel2 structure and domain boundary prediction by DISPORED and DOMPRED. Crosslinked amino acids L_{501} and K_{502} are highlighted in red.

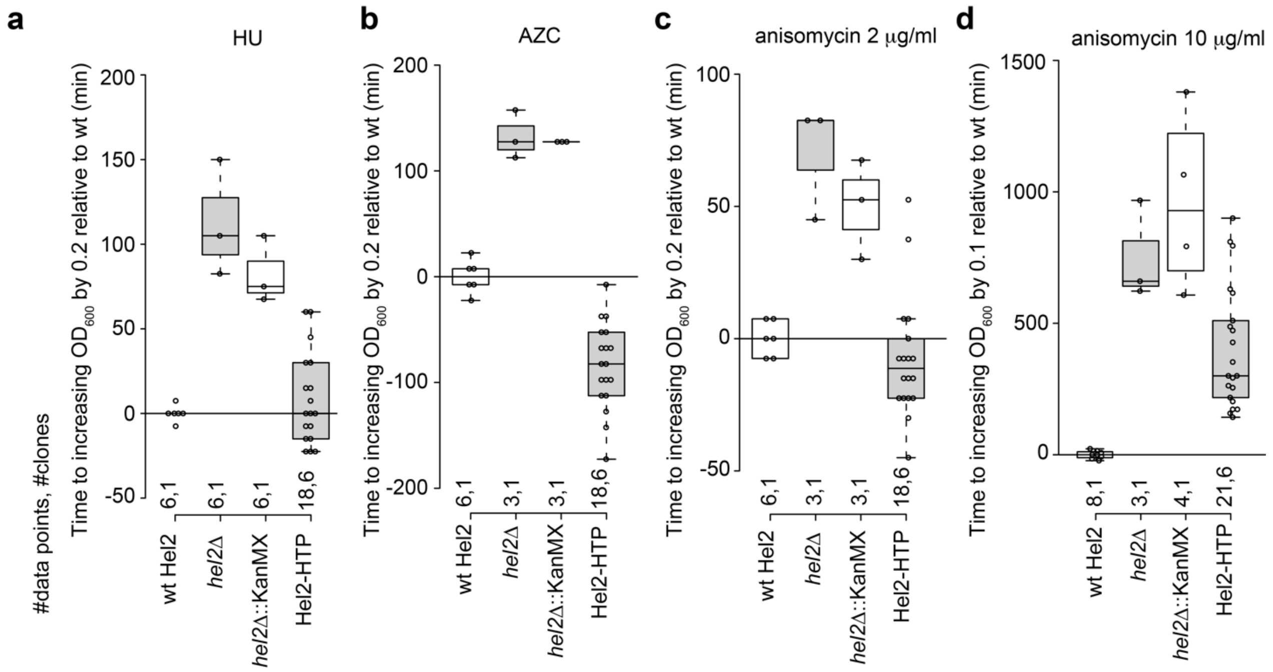

Supplementary Figure 4. Drug sensitivity of tagged and deletion strains as measured by plate-reader tests.

The graphs show the time required to increase OD_{600} by 0.2 (0.1 for 10 μg/ml anisomycin), as measured in plate-reader, with the wild-type (average of 2 samples/replicate) set to 0 min. Panels show sensitivity to **(a)** 50 mM hydroxyurea, **(b)** 50 μg/ml AZC, **(c)** 2 μg/ml anisomycin and **(d)** 10 μg/ml anisomycin. Centre lines of box plots show the medians; box limits indicate the 25th and 75th percentiles as determined by R software; whiskers extend 1.5 times the interquartile range from the 25th and 75th percentiles, outliers are represented by dots; data points are plotted as open circles. Number of sample points and number of clones used to generate those are shown as y-axis labels. Box plots were generated with http://shiny.chemgrid.org/boxplotr/

Strains lacking Hel2 were previously reported to be hypersensitive to each of the inhibitors[1-4] and this was confirmed for both *hel2*Δ strains. Growth of Hel2-HTP strains was comparable to wild-type in the presence of 50 mM HU **(a)** or 2 µg/ml anisomycin **(c)**. At high anisomycin concentrations (10 µg/ml), a mild growth impairment was observed for Hel2-HTP, but less than for *hel2*Δ **(d)**. In contrast, Hel2-HTP strains showed modest resistance to AZC **(b)**.
The mild effects of tagging may indicate that Hel2 has distinct functions in resolving the effects of different drugs.

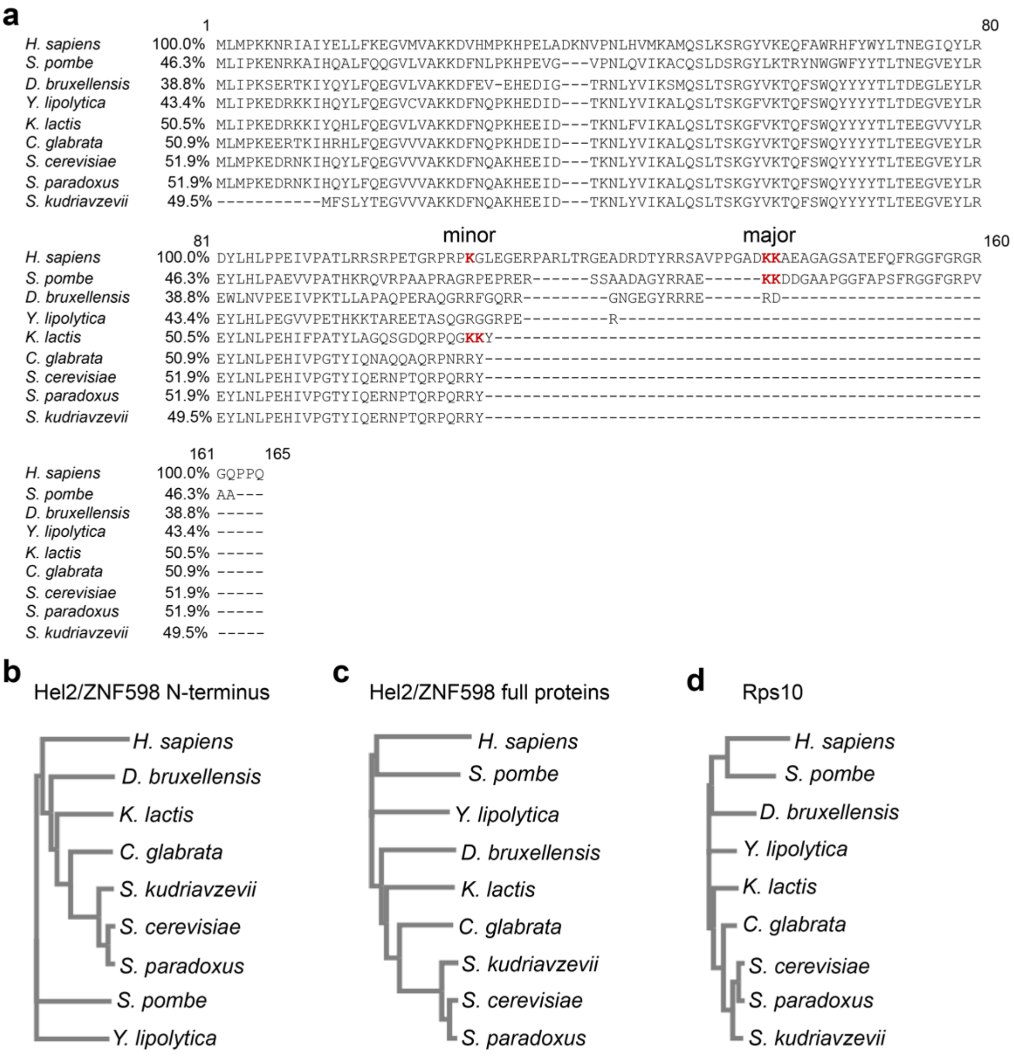

Supplementary Figure 5. Conservation of Rps10 and Hel2/ZNF598. **(a)** Multiple sequence alignment of Rps10 from human, fission yeast *S.pombe* and different budding yeast species. ZNF598 ubiquitination target lysine residues in *H. sapiens* and corresponding lysine residues in yeast species are highlighted in red. **(b-d)** Phylogenetic tree representation of conservation of, Hel2/ZNF598 N-termini **(b)**, full proteins **(c)** or Rps10 **(d).** Conservation of Hel2/ZNF598 full proteins mirror conservation of RPS10 to an extent, while N-termini alone do not follow this trend. The similarity between conservation of Hel2/ZNF598 full proteins and Rps10 must thus stem from the C-termini.

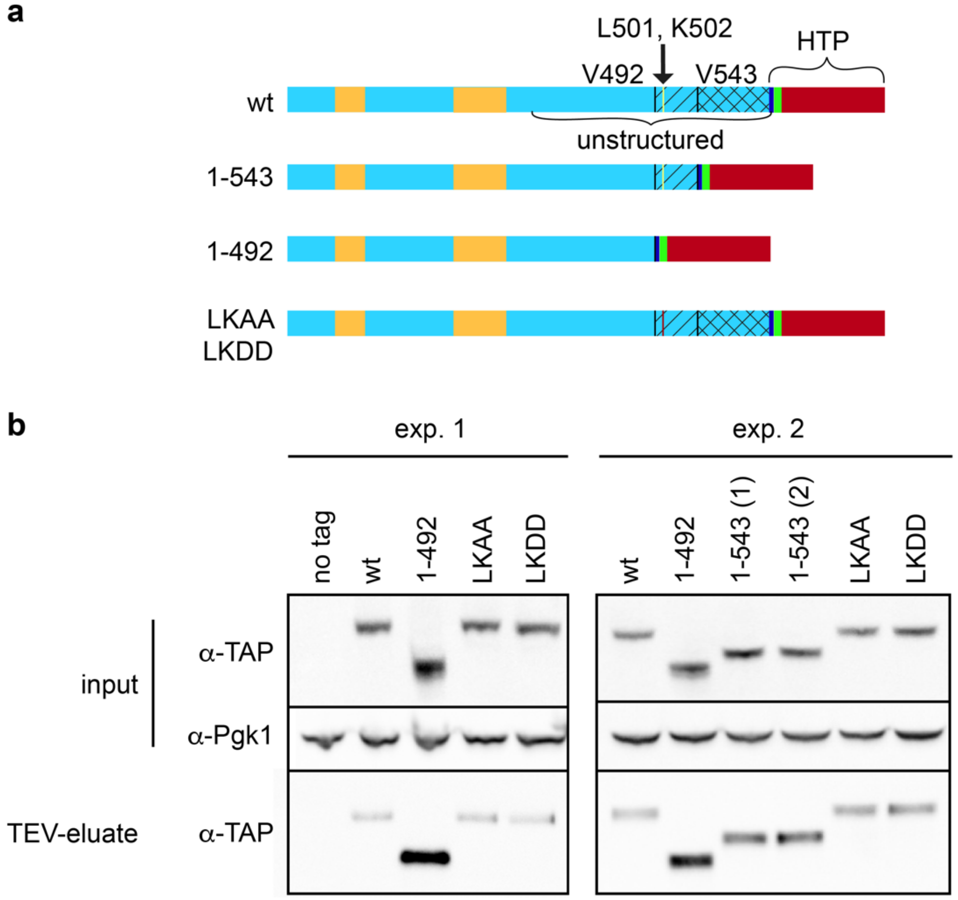

Supplementary Figure 6. (a) Schematic representation of Hel2 wild-type and mutants. Crosslinked amino acids L_{501} and K_{502} are highlighted. V_{492} and V_{543} are the new C-terminal positions of the two truncation mutants. V_{543} was chosen as a truncation position as A_{544} was identified as a potential domain boundary by DOMPRED prediction. **(b)** Western blot analysis of abundance of Hel2-HTP wild-type and mutant versions in input (lysates) and TEV-eluates. The truncated versions of Hel2-HTP were overexpressed, as seen in input, and also purified more efficiently than the wild-type versions, while point mutants behaved similar to wild-type.

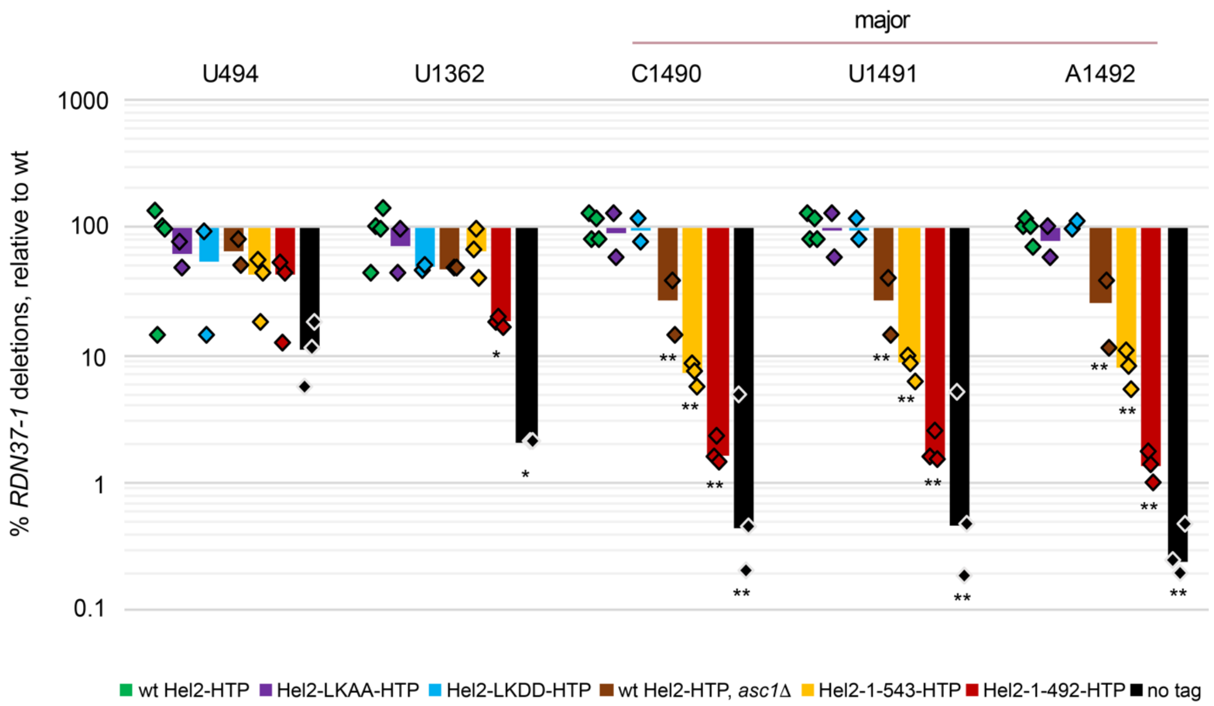

Supplementary Figure 7. Changes in deletions at minor and major 18S peaks for Hel2-HTP mutants or wild-type Hel2-HTP in *asc1Δ* background, relative to wild-type Hel2-HTP. % of deletions at peaks, relative to total deletions in *RDN37-1* in mutant strains were divided by median % deletion at peaks in wild-type strains. Bars show median, diamonds show single data points. Significance level was calculated by un-paired, 2-tailed, heteroscedastic Student's T-test (all samples of respective mutant against all wild-type samples). *: $p < 0.05$, **: $p < 0.005$. Related to Figures 4, 5.

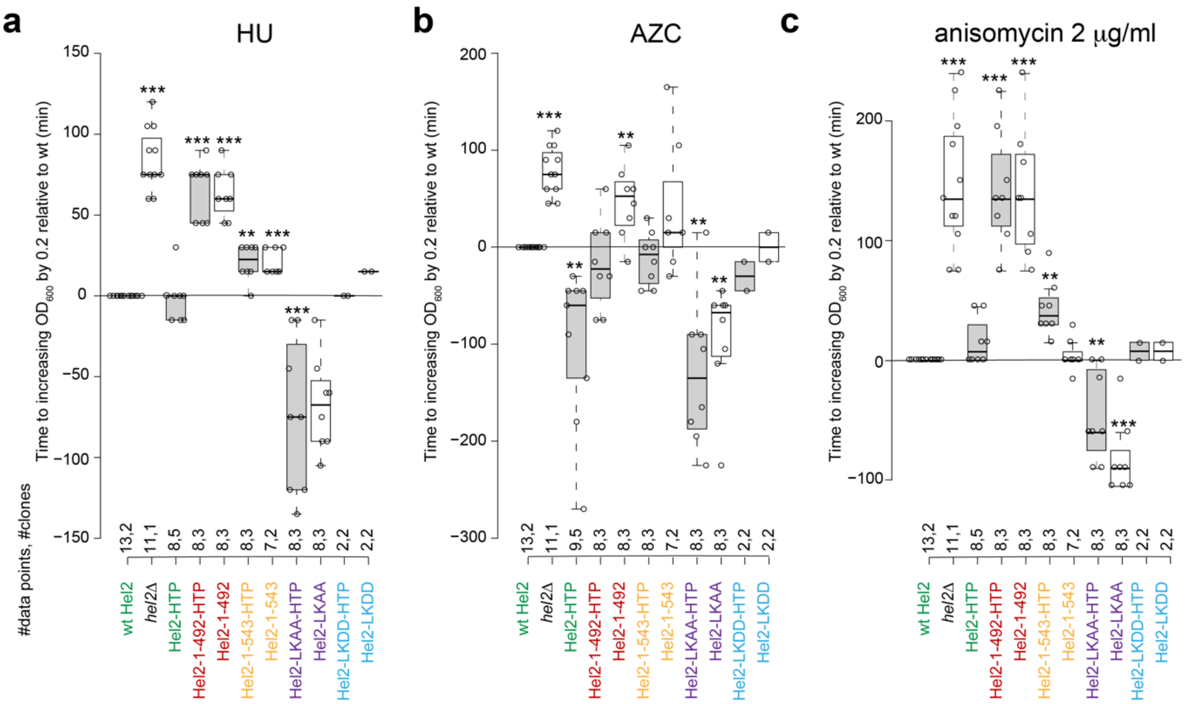

Supplementary Figure 8. Drug sensitivity to **(a)** 50 mM hydroxyurea, **(b)** 50 μg/ml AZC, and **(c)** 2 μg/ml anisomycin, as measured by plate-reader growth tests. The graphs show the time required to increase OD_{600} by 0.2, as measured in plate-reader, with the wild-type (1 sample/replicate) set to 0 min. HTP-tagged strains are represented with grey background. Positive values indicate hypersensitivity, negative values resistance. Center lines of box plots show the medians; box limits indicate the 25th and 75th percentiles as determined by R software; whiskers extend 1.5 times the interquartile range from the 25th and 75th percentiles, data points are plotted as open circles. Number of sample points and number of clones used for analysis are shown next to the y-axis. Box plots were generated with http://shiny.chemgrid.org/boxplotr/. *: $p < 0.05$, **: $p < 0.01$, ***: $p < 0.001$, w.r.t. wild-type Hel2, Student's T-test (2-tailed, paired, comparing each mutant sample with respective wild-type sample).

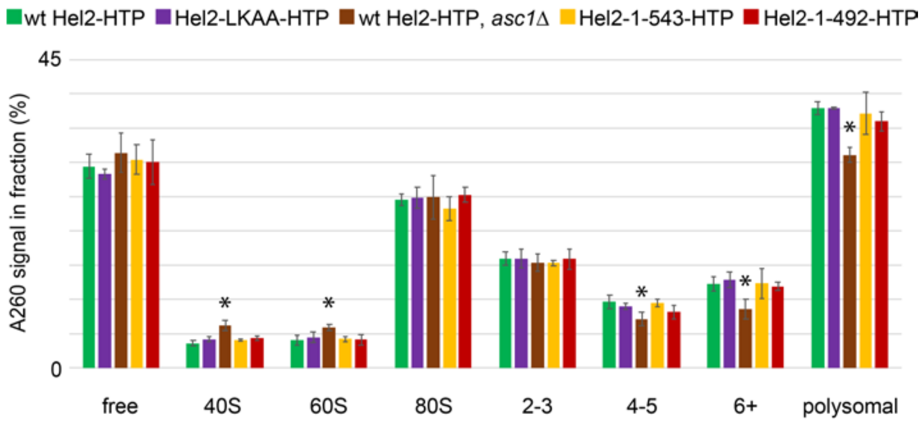

Supplementary Figure 9. Biological triplicate comparison of different fractions within the sucrose density gradients, based on A_{260} values for total RNA under different peaks. Error bars represent 1 S.D., * $p < 0.05$ in Student's T-test (2-tailed, paired, w.r.t. wild-type). Related to Figure 6.

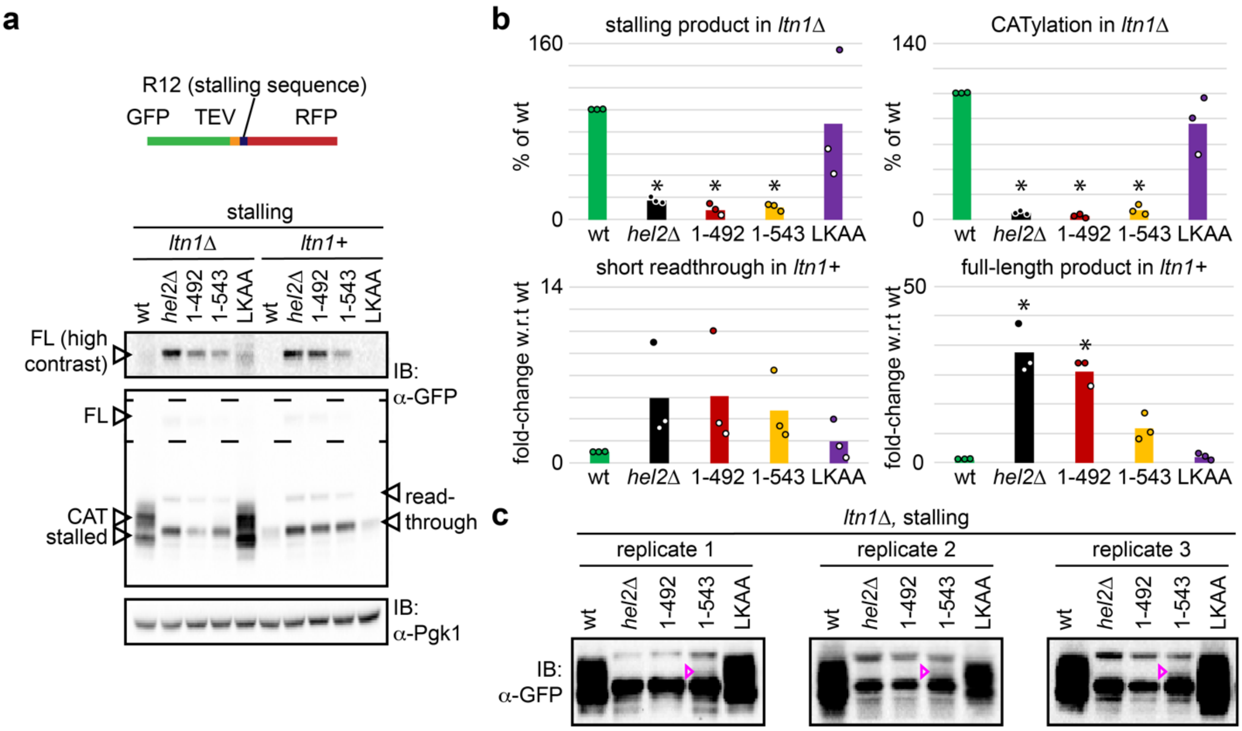

Supplementary Figure 10. Assessing RQC using a reporter construct and Western blot analysis. Related to Figure 7b. **(a)** Stalling construct (top, scheme adapted from ref.[5]) and representative Western blot (1 out of 3 biological replicates – individual clones). A high contrast version of the area in the dotted outline is shown in the top panel. The bottom panel shows the Pgk1 loading control. **(b)** Triplicate quantification of stalling product and CATylation product in *ltn1Δ* background and of short and full-length readthrough products in *ltn1*+ background. * $p < 0.05$ in Student's T-test (2-tailed, paired, w.r.t. the respective wild-type). **(c)** High contrast representation of the region containing stalled and CATylation products for *ltn1Δ* samples of all three replicates. A low-intensity band/smear (magenta-coloured arrow) of a size consistent with CATylation products in wild-type Hel2 and $Hel2_{LKAA}$ is seen for $Hel2_{1\text{-}543}$ but is not obvious for *hel2Δ* or $Hel2_{1\text{-}492}$. Accurate quantification of this band is difficult due to the more intense lower- and higher-running bands.

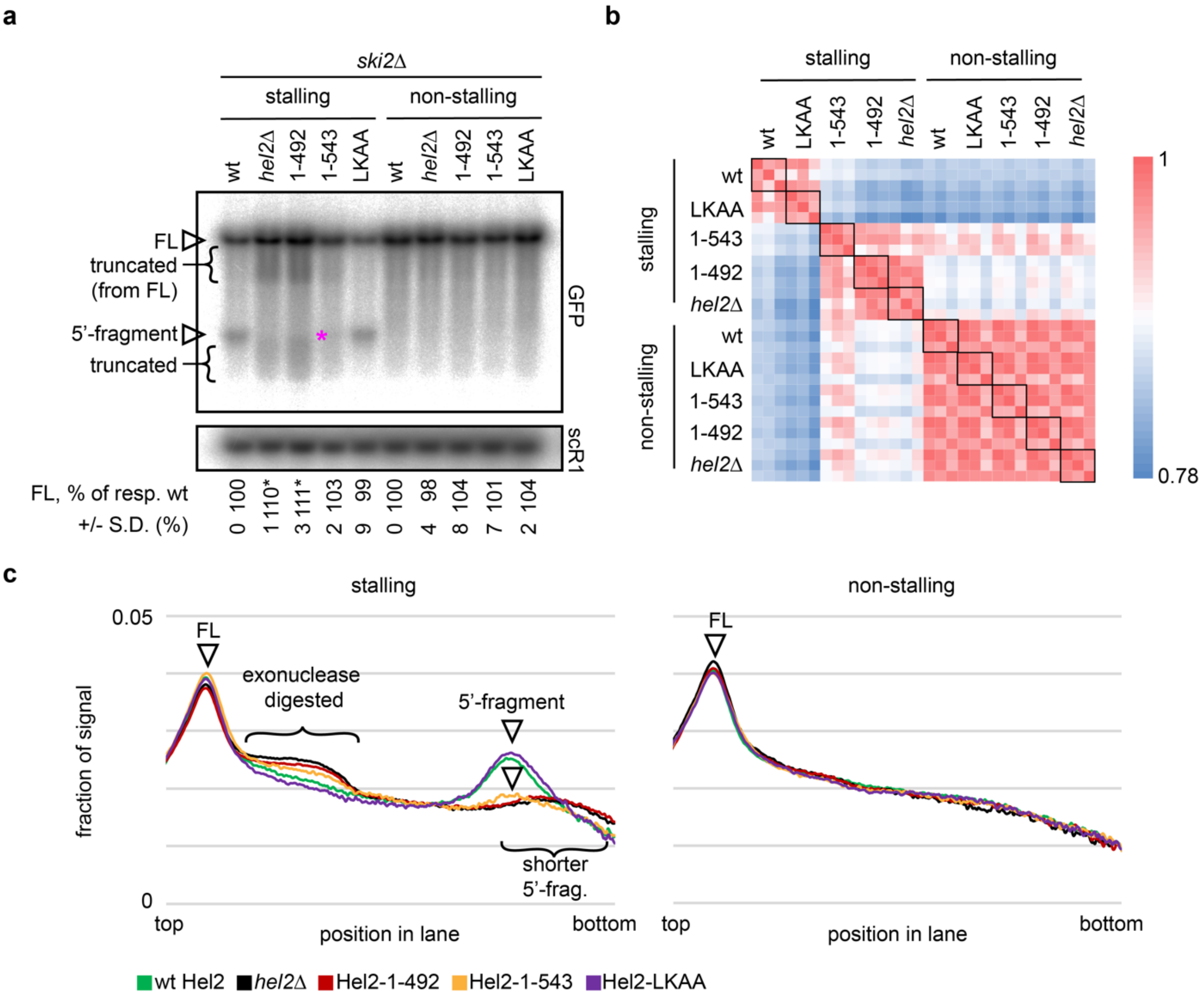

Supplementary Figure 11. Northern blot analysis of NGD using a reporter construct in different strains carrying different versions of Hel2 or *hel2Δ* (all in *ski2Δ* background, to allow for the 5'-fragment to be conserved.). Related to Figure 7c. **(a)** Representative autoradiograph showing Northern blot probed against GFP (probe annealing to the middle of the GFP part of the reporter) and against scR1 as a loading control. One of three biological replicates from three independent clones per strain is shown, as well as triplicate quantification (average ± S.D. of the full-length (FL) product after normalization to scR1 signal (except non-stalling LKAA, here n=2). * $p < 0.05$ in Student's T-test (2-tailed, paired, w.r.t. respective wild-type). Residual 5'-fragment is indicated for strain $Hel2_{1\text{-}543}$ (carrying stalling reporter) by a magenta-coloured star. **(b,c)** Analysis of intensities (fraction of signal after normalization to 1) over lanes containing different samples. **(b)** Pearson correlation between different samples of triplicate. Biological triplicates for each strain are boxed. **(c)** Average intensities over lanes for stalling (left) or non-stalling (right) reporter constructs. FL product, and canonical 5'-fragment (for wild-type, $Hel2_{LKAA}$ and $Hel2_{1\text{-}543}$ strains) are indicated by triangles. Truncated products are indicated by parentheses. In the strains carrying the stalling reporter, differences are seen in the pattern of bands/smears formed.

While strains carrying wild-type Hel2 or $Hel2_{LKAA}$ showed clear bands for the expected 5'-fragment, *hel2Δ* and $Hel2_{1\text{-}492}$ strains exhibited a lower-running smear instead of that band, as well as increased amounts of shorter sequences running below the FL product (see **a** and **c**). The strains expressing $Hel2_{1\text{-}543}$ showed and intermediate phenotype **(a,c)**, however correlating more with *hel2Δ* and $Hel2_{1\text{-}492}$ strains than with wild-type or $Hel2_{LKAA}$ **(b)**. There were no obvious differences seen between different strains when the non-stalling reporter was expressed **(a-c)**.

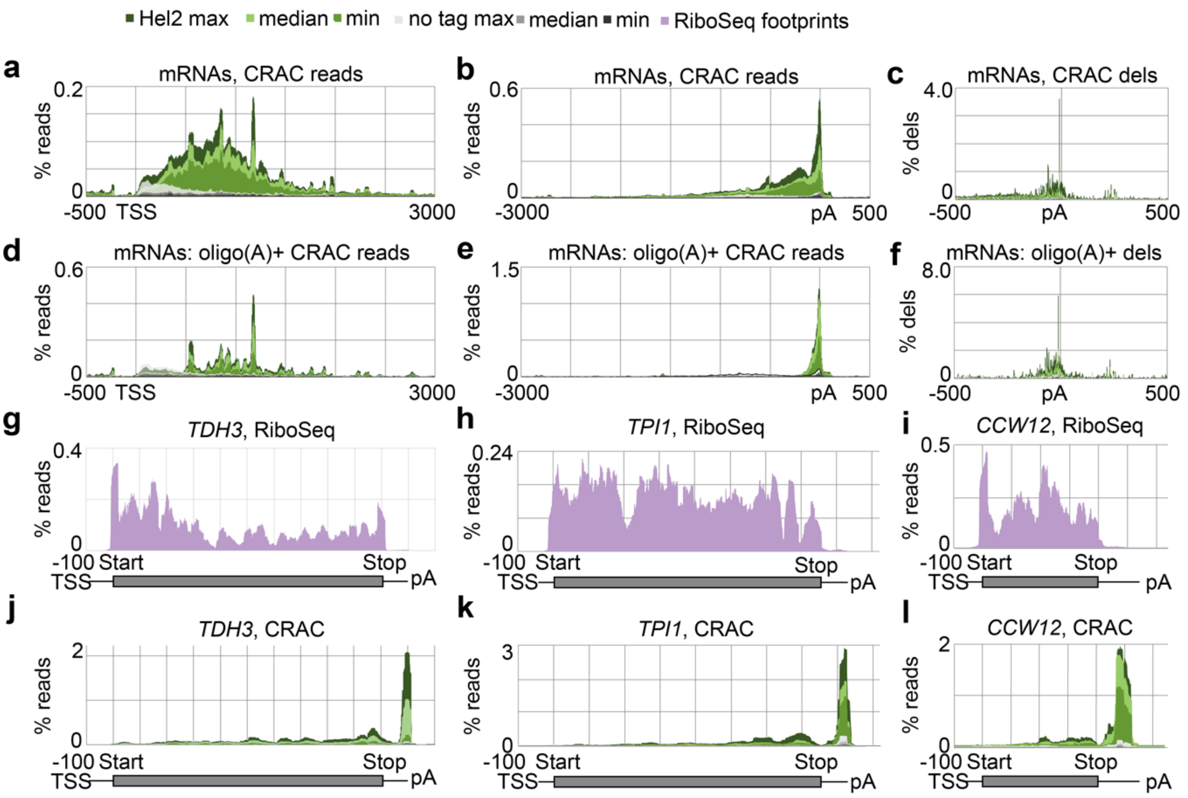

Supplementary Figure 12. Hel2 binding to mRNA. **(a-f)** Metagene analysis of collapsed datasets performed for the subset of top 275 reproducibly Hel2-bound protein-coding genes with mRNA length of ≥ 500 nt. **(a,b)** Total reads mapped across mRNA aligned by the annotated transcription start site (TSS) or polyadenylation site (pA). **(c)** Micro-deletions mapped across annotated pA sites. **(d,e)** Subset of sequences that contained a non-encoded poly(A) tail mapped across mRNA aligned by the annotated TSS or pA site. **(f)** Micro-deletions in reads with a non-encoded poly(A) tail, aligned by the annotated pA site. **(g-l)** RiboSeq footprints or Hel2 CRAC read distribution over three highly bound genes: **(g,j)** *TDH3*, **(h,k)** *TPI1,* **(i,l)** *CCW12*.

The values for "% reads" or "% deletions" shown are normalized to the sum of total Hel2 and no-tag signal, or to the sum of all RiboSeq footprints over the top reproducibly bound genes **(a-f)** or over individual genes **(g-l)**.

Legend is shown above panels.

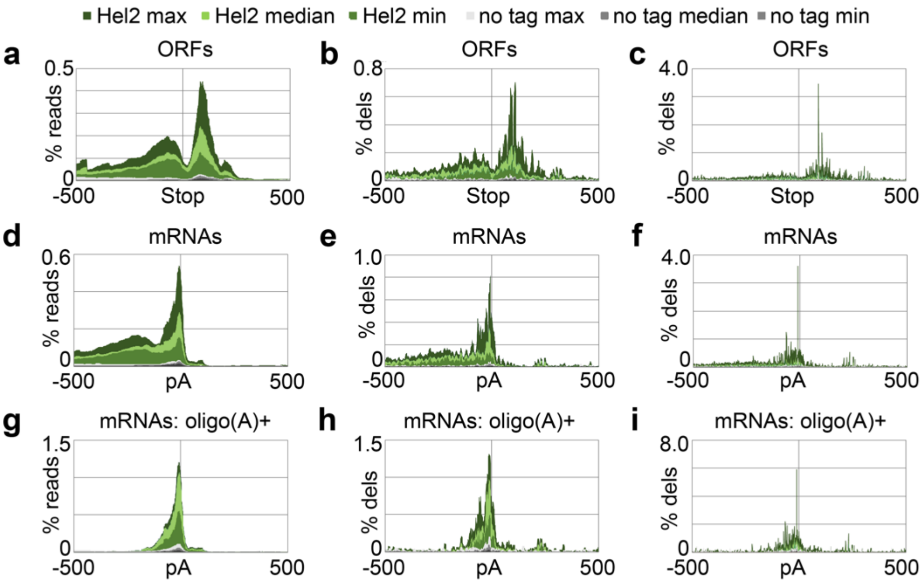

Supplementary Figure 13. Comparison between reads and deletions after and before smoothing. Analysis of 275 genes used in Figure 8b-g. ORFs aligned at stop codon **(a-c)**, complete mRNAs aligned at polyadenylation site (pA) **(d-f)** or mRNAs, considering only reads that contain non-templated oligo(A), aligned at pA **(g-i)**. **(a,d,g)** Read distribution. **(b,e,h)** Locations of micro-deletions after average-smoothing using a 9 nt sliding window. **(c,f,i)** Original deletions without smoothing. Legend is shown above panels. Related to Figure 8 and Supplementary Figure 12.

It is more evident that deletion patterns follow read patterns in the data after smoothing, than without smoothing.

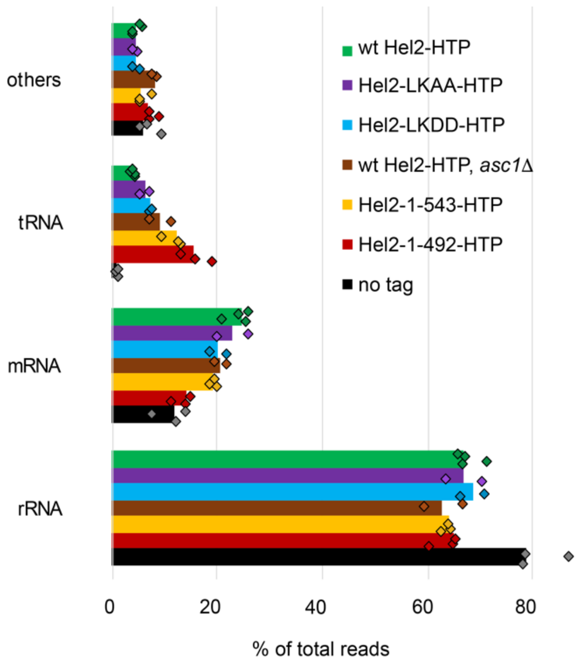

Supplementary Figure 14. Effect of Hel2 mutation or *ASC1* deletion on Hel2 crosslinking to different classes of RNA. Bars represent median, individual data points are added. Note that the relative abundance of RNA species for wild-type is not identical to the first set of experiments involving only wild-type Hel2-HTP, because the size fractionation of PAGE-separated protein-RNA complexes was adapted to cover the sizes of truncated as well as full-length versions of Hel2 leading to changes in percentage of types of RNA recovered.

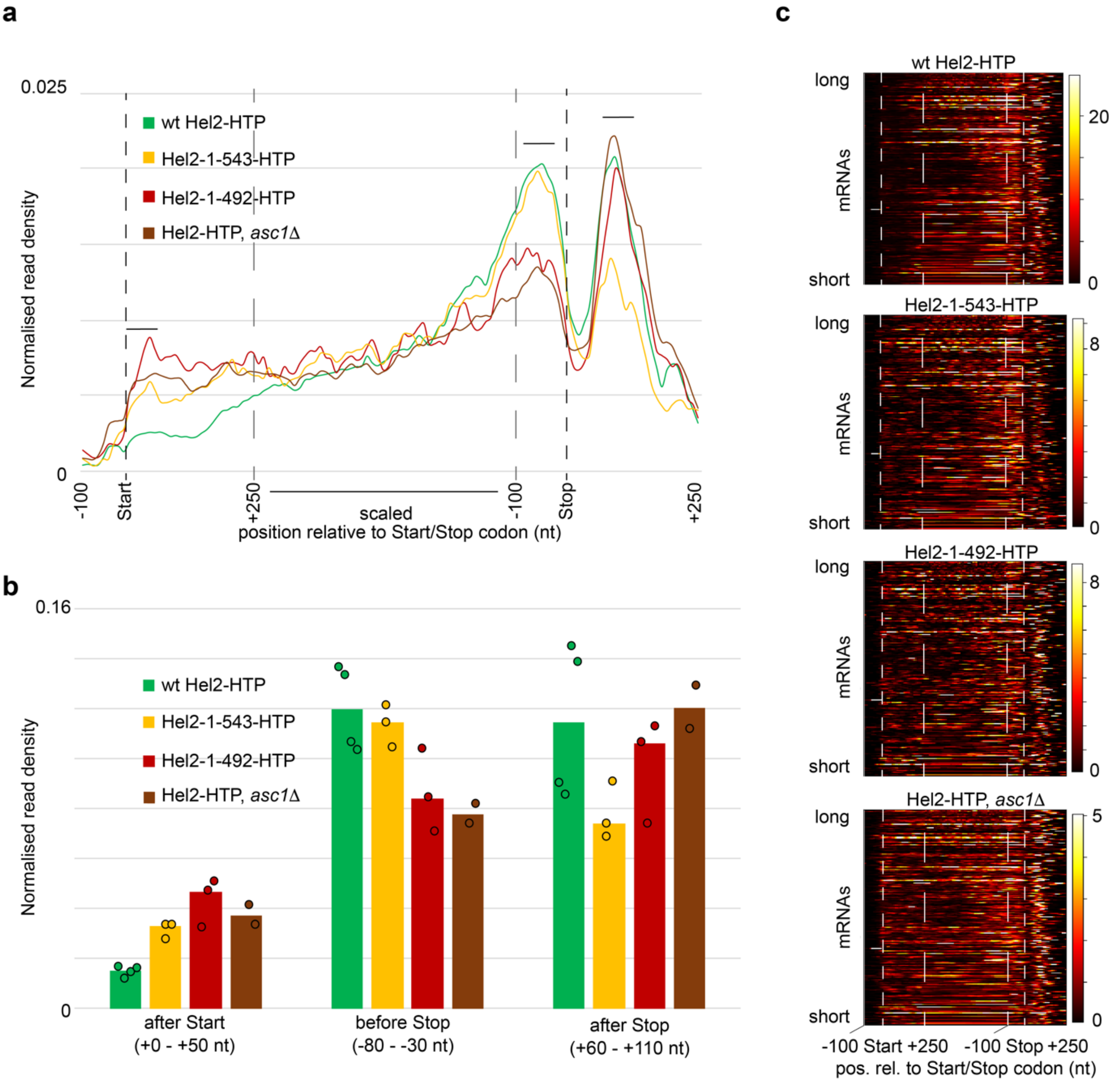

Supplementary Figure 15. Alternative quantification of data shown in Figure 9. Here, the area under each curve was normalized to 1 before calculating medians and signals are not shown relative to wild-type signal. Note that the total signal will be the same for all samples regardless of the crosslinking efficiency. **(a)** As Figure 9 b. **(b)** As Figure 9 c. **(c)** 2D plots aligned to start and stop codon positions (outer dashed lines), with nucleotides between start codon +250 nt and stop codon -100 nt (inner dashed lines) scaled. Data represent average of all available data sets for each strain. Before averaging, each data set was normalized to total read density of the corresponding wild-type data set for each technical replicate. mRNAs were scaled between start codon + 250 nt and stop codon -100 nt, as in panel **(a)**.

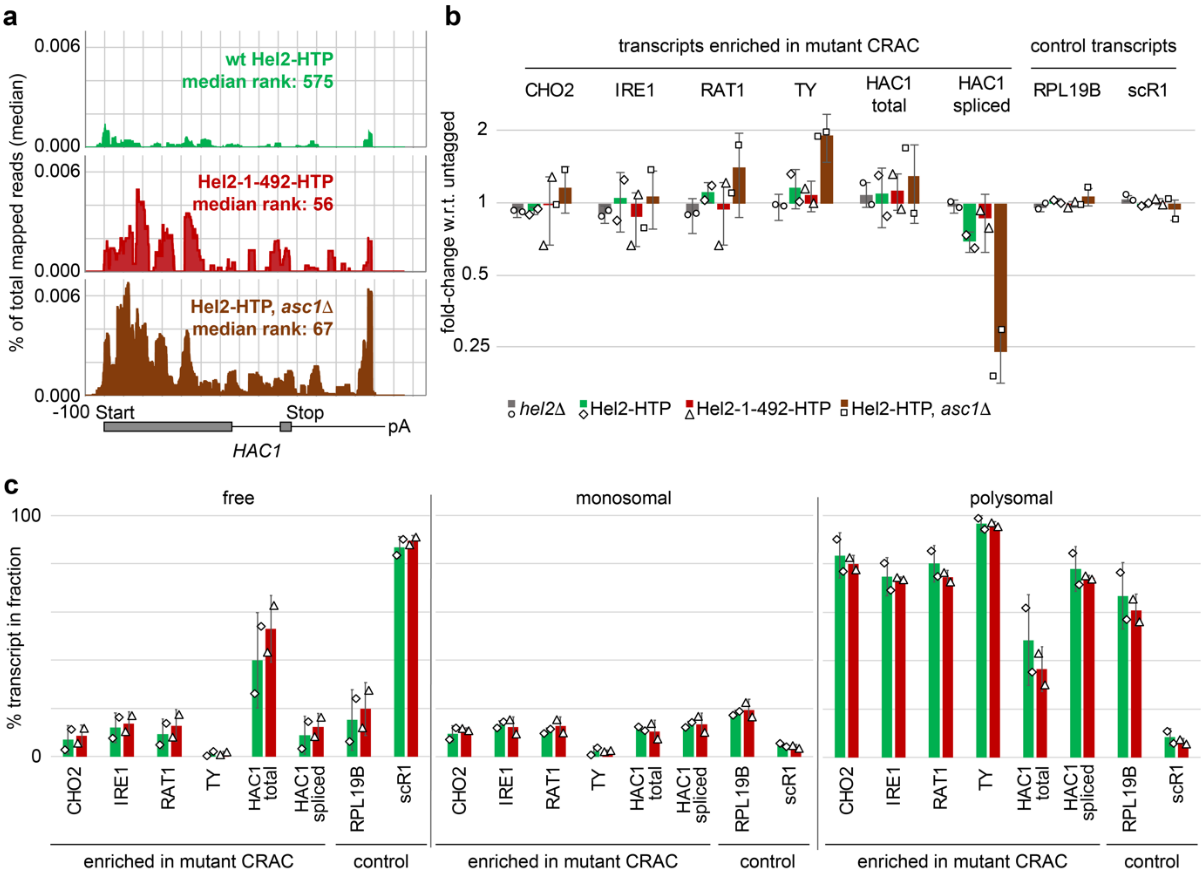

Supplementary Figure 16. Transcripts that were relatively enriched in CRAC for Hel2$_{1\text{-}492}$-HTP and Hel2-HTP, *asc1*Δ, compared to wild-type Hel2-HTP do not generally show increased mRNA or translation levels. **(a)** Read distribution over the HAC1 mRNA. Medians are shown for wild-type Hel2-HTP (n=4), Hel2$_{1\text{-}492}$-HTP (n=3) and Hel2-HTP, *asc1*Δ (n=2). All data are plotted as percentage of total mapped reads and median rank within the class of mRNAs is indicated. **(b)** qPCR was used to determine relative change in mRNA level for mRNAs enriched in CRAC datasets for Hel2$_{1\text{-}492}$-HTP and Hel2-HTP, *asc1*Δ, compared to wild-type Hel2-HTP. *hel2*Δ and different Hel2-HTP strains were compared to wild-type non-tagged strain. Fold-changes in enriched transcripts were normalized to the average changes of non-enriched transcripts RPL19B and scR1. **(c)** Distribution of enriched and non-enriched transcripts between free, monosomal and polysomal fraction in sucrose gradients, determined by qPCR. In **(b,c)** data represent average of 2 biological replicates. Each biological replicate was measured in technical triplicate. Error bars indicate S.D. Data points are shown. Legend for all panels in **(b)**.

Supplementary Table 1. Overview of samples and datasets used for wild-type Hel2-HTP CRAC and controls. See Supplementary Table 9 for more information on oligonucleotides.

Sample	barcode		indices	nr. fastq files processed
**exp.1 no tag**	L5_Ad	NNNCGCTTAGC	D03, D04	2
**exp.1 Hel2-HTP-1**	L5_Ca	NNNCTAGC	D03, D04	2
**exp.2 no tag**	L5_Eb	NNNGTGACAN	D05,D06	8
**exp.2 Hel2-HTP-2**	L5_Ed	NNNACAGTGN	D05,D06	8
**exp.3 no tag**	L5_Ba	NNNAGAGC	D09	1
**exp.3 Hel2-HTP-3**	L5_Bb	NNNGTGAGC	D09	1
**exp.3 Hel2-HTP-4**	L5_Bc	NNNCACTAGC	D09	1
**exp.4 no tag**	L5_Cc	NNNACTCAGC	D11	1
**exp.4 Hel2-HTP-5**	L5_Cd	NNNGACTTAGC	D11	1
**exp.4 Hel2-HTP-6**	L5_Da	NNNCGTGATN	D11	4

Supplementary Table 2. Numbers of aligned, uncollapsed reads after quality control in CRAC of wild-type Hel2-HTP and untagged control.

	exp.1	exp.2	exp.3	exp.4
**Hel2-HTP**	8,876,185	3,059,428	9,584,935	29,027,374
			12,937,126	18,669,585
**no tag**	1,544,568	1,481,193	1,017,142	5,471,666

Supplementary Table 3. Numbers of aligned, collapsed reads after quality control in CRAC of wild-type Hel2-HTP and untagged control.

	exp.1	exp.2	exp.3	exp.4
**Hel2-HTP**	788,139	575,198	691,727	1,960,775
			880,249	1,444,822
**no tag**	110,576	238,517	56,050	372,295

Supplementary Table 4. Class distribution of uncollapsed, mapped reads in CRAC of wild-type Hel2-HTP and untagged control. Visualized in Figure 2b.

	**rRNA**	**mRNA**	**tRNA**	**other**
**exp.1 no tag**	747,214	494,661	39,799	322,474
**exp.1 Hel2-HTP-1**	3,897,382	3,493,225	744,818	939,468
**exp.2 no tag**	754,214	442,807	25,097	315,483
**exp.2 Hel2-HTP-2**	1,649,800	936,600	116,422	442,861
**exp.3 no tag**	600,512	198,108	21,199	220,824
**exp.3 Hel2-HTP-3**	4,005,256	4,306,997	688,593	831,964
**exp.3 Hel2-HTP-4**	7,336,430	3,892,009	769,654	1,156,549
**exp.4 no tag**	3,263,076	1,318,312	136,593	912,801
**exp.4 Hel2-HTP-5**	16,979,387	9,069,396	1,870,047	1,647,232
**exp.4 Hel2-HTP-6**	12,142,194	5,153,334	803,621	867,132

Supplementary Table 5. Overview of samples and datasets used for the study of mutant Hel2-HTP versions by CRAC. See Supplementary Table 9 for more information on oligonucleotides.

Sample	barcode		indices	nr. fastq files processed
**exp.1 no tag**	L5_Ba	NNNAGAGC	D01, D02 (low fraction); D03, D04 (high fraction)	4
**exp.1 Hel2-HTP**	L5_Bb	NNNGTGAGC	D01, D02 (low fraction); D03, D04 (high fraction)	4
**exp.1 Hel2$_{1-492}$-HTP**	L5_Bc	NNNCACTAGC	D01, D02 (low fraction); D03, D04 (high fraction)	4
**exp.1 Hel2$_{LKAA}$-HTP**	L5_Bd	NNNTCTCTAGC	D01, D02 (low fraction); D03, D04 (high fraction)	4
**exp.1 Hel2$_{LKDD}$-HTP**	L5_Ca	NNNCTAGC	D01, D02 (low fraction); D03, D04 (high fraction)	4
**exp.2 Hel2-HTP**	L5_Cc	NNNACTCAGC	D07 (low fraction); D08 (high fraction)	2
**exp.2 Hel2$_{1-492}$-HTP**	L5_Cd	NNNGACTTAGC	D07 (low fraction); D08 (high fraction)	8
**exp.2 Hel2$_{1-543}$-HTP (1)**	L5_Da	NNNCGTGATN	D07 (low fraction); D08 (high fraction)	8
**exp.2 Hel2$_{1-543}$-HTP (2)**	L5_Db	NNNGCACTAN	D07 (low fraction); D08 (high fraction)	8
**exp.2 Hel2$_{LKAA}$-HTP**	L5_Dc	NNNTAGTGCN	D07 (low fraction); D08 (high fraction)	8
**exp.2 Hel2$_{LKDD}$-HTP**	L5_De	NNNATCACGN	D07 (low fraction); D08 (high fraction)	8
**exp.3 no tag**	L5_Ed	NNNACAGTGN	D09, D10	8
**exp.3 Hel2-HTP**	L5_Aa	NNNTAAGC	D09, D10	2
**exp.3 Hel2$_{1-492}$-HTP**	L5_Ab	NNNATTAGC	D09, D10	2
**exp.3 Hel2$_{1-543}$-HTP**	L5_Ac	NNNGCGCAGC	D09, D10	2
**exp.3 Hel2-HTP *asc1Δ***	L5_Ad	NNNCGCTTAGC	D09, D10	2
**exp.4 no tag**	L5_Bb	NNNGTGAGC	D11, D12	2
**exp.4 Hel2-HTP**	L5_Bc	NNNCACTAGC	D11, D12	2
**epx.4 Hel2-HTP *asc1Δ***	L5_Bd	NNNTCTCTAGC	D11, D12	2

Supplementary Table 6. Numbers of aligned, uncollapsed reads after quality control in CRAC of Hel2-HTP mutants and wild-type Hel2-HTP in *asc1Δ* background.

	**exp.1**	**exp.2**		**exp.3**	**exp.4**
**wt**	3,216,107	4,880,511		4,248,380	5,883,685
**LKAA**	3,441,390	863,776			
**LKDD**	4,355,353	3,425,388			
***asc1*Δ**				1,008,794	3,911,276
**1-543**		1,174,780	3,010,253	2,103,985	
**1-492**	4,129,544	2,404,848		3,563,976	
**no tag**	191,529			1,064,107	602,841

Supplementary Table 7. Numbers of aligned, collapsed reads after quality control in CRAC of Hel2-HTP mutants and wild-type Hel2-HTP in *asc1Δ* background.

	**exp.1**	**exp.2**		**exp.3**	**exp.4**
**wt**	354,392	567,051		735,704	1,631,374
**LKAA**	413,649	144,864			
**LKDD**	508,036	441,794			
***asc1*Δ**				198,444	1,064,852
**1-543**		168,654	422,502	342,350	
**1-492**	427,385	241,385		546,773	
**no tag**	28,537			151,365	156,449

Supplementary Table 8. Strains used in this study.

Strain	Genotype	Parental strain.	Nr. of clones	Reference or source
**BY4741**	*MATa, his3Δ, leu2Δ, met15Δ, ura3Δ*	-	2	Brachmann *et al.*[6]
**Hel2-HTP**	BY4741, Hel2-HTP::URA3	BY4741	6	This study.
***hel2*Δ**	BY4741, *hel2*Δ (*delitto perfetto*, using the 50:50 method described in ref[7])	BY4741	1	This study.
***hel2*Δ::KanMX**	BY4741, *hel2*Δ::KanMX	BY4741	1	This study.
**Hel2$_{1\text{-}492}$**	BY4741, Hel2$_{1\text{-}492}$	Hel2-HTP	3	This study.
**Hel2$_{1\text{-}492}$-HTP**	BY4741, Hel2$_{1\text{-}492}$-HTP::URA3	BY4741	3	This study.
**Hel2$_{1\text{-}543}$**	BY4741, hel2$_{1\text{-}543}$	Hel2-HTP	2	This study.
**Hel2$_{1\text{-}543}$-HTP**	BY4741, Hel2$_{1\text{-}543}$-HTP::URA3	BY4741	3	This study.
**Hel2$_{LKAA}$**	BY4741, Hel2$_{L501AK502A}$	Hel2-1-492-HTP	3	This study.
**Hel2$_{LKAA}$-HTP**	BY4741, Hel2$_{L501AK502A}$-HTP::URA3	Hel2-1-492	3	This study.
**Hel2$_{LKDD}$**	BY4741, Hel2$_{L501DK502D}$	Hel2-1-492-HTP	2	This study.
**Hel2$_{LKDD}$-HTP**	BY4741, Hel2$_{L501DK502D}$-HTP::URA3	Hel2-1-492	2	This study.
**Hel2-HTP, *asc1*Δ**	BY4741, *asc1*Δ::KanMX, Hel2-HTP::URA3	Hel2-HTP	3	This study.
***ltn1*Δ::KanMX**	BY4741, *ltn1*Δ::KanMX	BY4741	1	This study.
***hel2*Δ, *ltn1*Δ::KanMX**	BY4741, *hel2*Δ (*delitto perfetto*) , ltn1Δ::KanMX	*hel2*Δ	1	This study.
**Hel2$_{1\text{-}492}$, *ltn1*Δ::KanMX**	BY4741, Hel2$_{1\text{-}492}$, *ltn1*Δ::KanMX	Hel2$_{1\text{-}492}$	1	This study.
**Hel2$_{1\text{-}543}$, *ltn1*Δ::KanMX**	BY4741, Hel2$_{1\text{-}543}$, *ltn1*Δ::KanMX	Hel2$_{1\text{-}543}$	1	This study.
**Hel2$_{LKAA}$, *ltn1*Δ::KanMX**	BY4741, Hel2$_{L501AK502A}$, *ltn1*Δ::KanMX	Hel2$_{LKAA}$	1	This study.
***ski2*Δ::KanMX**	BY4741, *ski2*Δ::KanMX	BY4741	1	This study.
***hel2*Δ, *ski2*Δ::KanMX**	BY4741, *hel2*Δ (*delitto perfetto*) , *ski2*Δ::KanMX	*hel2*Δ	1	This study.
**Hel2$_{1\text{-}492}$, *ski2*Δ::KanMX**	BY4741, Hel2$_{1\text{-}492}$, *ski2*Δ::KanMX	Hel2$_{1\text{-}492}$	1	This study.
**Hel2$_{1\text{-}543}$, *ski2*Δ::KanMX**	BY4741, Hel2$_{1\text{-}543}$, *ski2*Δ::KanMX	Hel2$_{1\text{-}543}$	1	This study.
**Hel2$_{LKAA}$, *ski2*Δ::KanMX**	BY4741, Hel2$_{L501AK502A}$, *ski2*Δ::KanMX	Hel2$_{LKAA}$	1	This study.
**BY4741, pGTRR**	BY4741, pGTRR[5]	BY4741	3	This study.

hel2*Δ, *pGTRR	BY4741, *hel2*Δ (*delitto perfetto*) , pGTRR[5]	*hel2*Δ	3	This study.
**Hel2$_{1-492}$, pGTRR**	BY4741, Hel2$_{1-492}$, pGTRR[5]	Hel2$_{1-492}$	3	This study.
**Hel2$_{1-543}$, pGTRR**	BY4741, Hel2$_{1-543}$, pGTRR[5]	Hel2$_{1-543}$	3	This study.
**Hel2$_{LKAA}$, pGTRR**	BY4741, Hel2$_{L501AK502A}$, pGTRR[5]	Hel2$_{LKAA}$	3	This study.
***ltn1*Δ::KanMX, pGTRR**	BY4741, *ltn1*Δ::KanMX, pGTRR[5]	*ltn1*Δ::KanMX	3	This study.
***hel2*Δ, *ltn1*Δ::KanMX, pGTRR**	BY4741, *hel2*Δ (*delitto perfetto*) , *ltn1*Δ::KanMX, pGTRR[5]	*hel2*Δ, *ltn1*Δ::KanMX	3	This study.
**Hel2$_{1-492}$, *ltn1*Δ::KanMX, pGTRR**	BY4741, Hel2$_{1-492}$, *ltn1*Δ::KanMX, pGTRR[5]	Hel2$_{1-492}$, *ltn1*Δ::KanMX	3	This study.
**Hel2$_{1-543}$, *ltn1*Δ::KanMX, pGTRR**	BY4741, Hel2$_{1-543}$, *ltn1*Δ::KanMX, pGTRR[5]	Hel2$_{1-543}$, *ltn1*Δ::KanMX	3	This study.
**Hel2$_{LKAA}$, *ltn1*Δ::KanMX, pGTRR**	BY4741, Hel2$_{L501AK502A}$, *ltn1*Δ::KanMX, pGTRR[5]	Hel2$_{LKAA}$, *ltn1*Δ::KanMX	3	This study.
***ski2*Δ::KanMX, pGTRR**	BY4741, *ski2*Δ::KanMX, pGTRR[5]	*ski2*Δ::KanMX	3	This study.
***hel2*Δ, *ski2*Δ::KanMX, pGTRR**	BY4741, *hel2*Δ (*delitto perfetto*) , *ski2*Δ::KanMX, pGTRR[5]	*hel2*Δ, *ski2*Δ::KanMX	3	This study.
**Hel2$_{1-492}$, *ski2*Δ::KanMX, pGTRR**	BY4741, Hel2$_{1-492}$, ski2Δ::KanMX, pGTRR[5]	Hel2$_{1-492}$, *ski2*Δ::KanMX	3	This study.
**Hel2$_{1-543}$, *ski2*Δ::KanMX, pGTRR**	BY4741, Hel2$_{1-543}$, ski2Δ::KanMX, pGTRR[5]	Hel2$_{1-543}$, *ski2*Δ::KanMX	3	This study.
**Hel2$_{LKAA}$, *ski2*Δ::KanMX, pGTRR**	BY4741, Hel2$_{L501AK502A}$, *ski2*Δ::KanMX, pGTRR[5]	Hel2$_{LKAA}$, *ski2*Δ::KanMX	3	This study.
***ski2*Δ::KanMX, pGTSTR**	BY4741, *ski2*Δ::KanMX, pGTSTR[5]	*ski2*Δ::KanMX	3	This study.
***hel2*Δ, *ski2*Δ::KanMX, pGTSTR**	BY4741, *hel2*Δ (*delitto perfetto*) , *ski2*Δ::KanMX, pGTSTR[5]	*hel2*Δ, *ski2*Δ::KanMX	3	This study.
**Hel2$_{1-492}$, *ski2*Δ::KanMX, pGTSTR**	BY4741, Hel2$_{1-492}$, *ski2*Δ::KanMX, pGTSTR[5]	Hel2$_{1-492}$, *ski2*Δ::KanMX	3	This study.
**Hel2$_{1-543}$, *ski2*Δ::KanMX, pGTSTR**	BY4741, Hel2$_{1-543}$, *ski2*Δ::KanMX, pGTSTR[5]	Hel2$_{1-543}$, *ski2*Δ::KanMX	3	This study.
**Hel2$_{LKAA}$, *ski2*Δ::KanMX, pGTSTR**	BY4741, Hel2$_{L501AK502A}$, *ski2*Δ::KanMX, pGTSTR[5]	Hel2$_{LKAA}$, *ski2*Δ::KanMX	3	This study.

Supplementary Table 9. Oligonucleotides used in this study. Variable regions of 5'-linkers and indexing primers are underlined.

Name	Sequence	Use
**Tagging, mutation, deletion and test primers**		
**Ptag_Hel2-HTP_f**	5'-AAAAAGAAAGGCAAACAAAAGCA GCTGTTATTCCACATTGGTGTAGAGC ACCATCACCATCACC-OH-3'	HTP-tagging Hel2, deletion of *HEL2* by 50:50 method[7]
**Ptag_Hel2-HTP_r**	5'-TTCTCTAATGCTATTGTCAGTTAC AGGTTAGAAATATATTTCCAATACGA CTCACTATAGGG-OH-3'	HTP-tagging Hel2
**Ptest_HTP-in_f1**	5'-CCCTATAGTGAGTCGTA-OH-3'	testing tagging or deletion of Hel2
**Ptest_HTP-in_f2**	5'-TATGATTGTCTCCGGG-OH-3'	testing tagging or deletion of Hel2
**Ptest_ Hel2-in-r1**	5'-CGTCTGAGCAGGAGA-OH-3'	testing tagging or deletion of Hel2, also used to generate and test point-mutants
**Ptest_ Hel2in-r2**	5'-CGGCAATAGAGGAACTAA-OH-3'	testing tagging or deletion of Hel2
**Ptest_ Hel2-in-f1**	5'-GTCGAGCGCATACCTA-OH-3'	testing tagging or deletion of Hel2
**Pdel_ *HEL2*-in2r-outr-HTPr**	5'-TTCATTTCTCTAATGCTATTGTCAG TTACAGGTTAGAAATATATTTCCAAC CTAATTCTTGAAAAGAAGTATAGCCA CTATTTTTTCGACGAAAGAGACTTAC GACTCACTATAGGG-OH-3'	deletion of *HEL2* by 50:50 method
**Pdel_ *HEL2*-F1-pFA6a-KanMX**	5'-AGTCTCTTTCGTCGAAAAAATAGT GGCTATACTTCTTTTCAAGAATTAGG CGGATCCCCGGGTTAATTAAG-OH-3'	deletion of *HEL2* by exchange with KanMX cassette
**Pdel_*HEL2*-R-pFA6a-KanMX**	5'-TTCATTTCTCTAATGCTATTGTCAG TTACAGGTTAGAAATATATTTCCAAG AATTCGAGCTCGTTTAAAC-OH-3'	deletion of *HEL2* by exchange with KanMX cassette
**Pmut_ Hel2_1-492_HTP_f**	5'-GTGGAACAAACGATGGGGGCAG CGCTGGTGCAGCATTAGGCGTTGAG CACCATCACCATC-OH-3'	generating HTP-tagged truncation mutant of Hel2
**Pmut_ Hel2_1-543_HTP_f**	5'-CTACACCTCAATCAGTTAGTTATC GAACTTCCACTAATACAGTCGAGCAC CATCACCATC-OH-3'	generating HTP-tagged truncation mutant of Hel2
**Pmut_ Hel2_1-492_f**	5'-GGTGGAACAAACGATGGGGGCA GCGCTGGTGCAGCATTAGGCGTTTA GTTGGAA-OH-3'	primer extension with reverse primer, to generate non-tagged truncation mutant
**Pmut_ Hel2_1-492_r**	5'-TCTAATGCTATTGTCAGTTACAGG TTAGAAATATATTTCCAACTAAACGC CTAA-OH-3'	primer extension with forward primer, to generate non-tagged truncation mutant
**Pmut_ Hel2_1-543_f**	5'-ACTACACCTCAATCAGTTAGTTAT CGAACTTCCACTAATACAGTCTAGTT GGAA-OH-3'	primer extension with reverse primer, to generate non-tagged truncation mutant
**Pmut_Hel2_1-543_r**	5'-TCTAATGCTATTGTCAGTTACAGG TTAGAAATATATTTCCAACTAGACTG TATT-OH-3'	primer extension with forward primer, to generate non-tagged truncation mutant
**Pmut_Hel2_LKAA _f**	5'-AGGAATATCAAAAATCTTCCAACC GCGGCAAGTCCTTCAGCGTCG-OH-3'	generating LKAA point mutant
**Pmut_Hel2_LKDD _f**	5'-AGGAATATCAAAAATCTTCCAACC GATGACAGTCCTTCAGCGTCG-OH-3'	generating LKDD point mutant
**Pmut_LKNN-5ext1-f**	5'-AACGATGGGGGCAGCGCTGGTG CAGCATTAGGCGTTAGGAATATCAAA AATCTTCCAACC-OH-3'	primer extension, to generate point mutants
**Ptest_ Hel2-mut-del-f**	5'-TGTTATTCACAAAACCAAAT GC-OH-3'	testing point and truncation mutants
**Ptest_Hel2-mut-del-r**	5'-GTGTTCAATTCCCTCAAATTA TC-OH-3'	testing point and truncation mutants
**Pdel_ *ASC1*-KanMX_F**	5'-AAATCCTTATAACACACTAAAGTA AATAAAGTGAAAAATGCGGATCCCC GGGTTAATTAA-OH-3'	deletion of *ASC1* by exchange with KanMX cassette
**Pdel_ *ASC 1*-KanMX_R**	5'-ACATAAAAGAACAAATGAACTTTA TACATATTCTTAGTTAGAATTCGAGC TCGTTTAAAC-OH-3'	deletion of *ASC1* by exchange with KanMX cassette
**Ptest_ *ASC 1*-in-f1**	5'-ACTGCTCCTTTGGTTTTC-OH-3'	testing deletion of *ASC 1*
**Ptest_ *ASC 1*-in-r1**	5'-GTATTTTCGCAGCAAACAG-OH-3'	testing deletion of *ASC 1*

**Pdel_ *LTN1*-KanMX_F**	5'-AGCCATCAAAAAAAGTTCAAGCAATAGTTGGTTCTTAATGCGGATCCCCGGGTTAATTAA-OH-3'	deletion of *LTN1* by exchange with KanMX cassette
**Pdel_ *LTN 1*-KanMX_R**	5'-AAAAATGTAGTACATTTATATGAAATTTATATGCGATAGTGAATTCGAGCTCGTTTAAAC-OH-3'	deletion of *LTN1* by exchange with KanMX cassette
**Ptest_ *LTN 1*-in-f1**	5'-CTTGTCAATTCGAGTTAACCC-OH-3'	testing deletion of *LTN1*
**Ptest_ *LTN 1*-in-r1**	5'-CAAGGTATAGGGCTGG-OH-3'	testing deletion of *LTN1*
**Pdel_*SKI2*-KanMX_F**	5'-CTAACTCACAAAATTTACTGTACTAATACTAATTTATATGCGGATCCCCGGGTTAATTAA-OH-3'	deletion of *SKI2* by exchange with KanMX cassette
**Pdel_ *SKI 2*-KanMX_R**	5'-CTTTTATAAACATGACTCACATTGAGAATAAATGAGCTCTGAATTCGAGCTCGTTTAAAC-OH-3'	deletion of *SKI2* by exchange with KanMX cassette
**Ptest_ *SKI 2*-in-f1**	5'-GTTAATGATATCACGACGGAC-OH-3'	testing deletion of *SKI2*
**Ptest_ *SKI 2*-in-r1**	5'-CATATTAGTTTATGTGATGAGGC-OH-3'	testing deletion of *SKI2*
**Ptest_KanMX-in-f1 (pFAchk1)**	5'-TTTCGCCTCGACATCATCTG-OH-3'	testing deletions by testing insertion of KanMX cassette together with reverse gene-specific primer
	**Library construction**	
**L3**	5'-rAppTGGAATTCTCGGGTGCCAAGG-ddC-3'	3'-linker
**L5Aa**	5'-invddT-ACACrGrArCrGrCrUrCrUrUrCrCrGrArUrCrUrNrNrNrUrArArGrC-OH-3'	5'-linker
**L5Ab**	5'-invddT-ACACrGrArCrGrCrUrCrUrUrCrCrGrArUrCrUrNrNrNrArUrUrArGrC-OH-3'	5'-linker
**L5Ac**	5'-invddT-ACACrGrArCrGrCrUrCrUrUrCrCrGrArUrCrUrNrNrNrGrCrGrCrArGrC-OH-3'	5'-linker
**L5Ad**	5'-invddT-ACACrGrArCrGrCrUrCrUrUrCrCrGrArUrCrUrNrNrNrCrGrCrUrUrArGrC-OH-3'	5'-linker
**L5Ba**	5'-invddT-ACACrGrArCrGrCrUrCrUrUrCrCrGrArUrCrUrNrNrNrArGrArGrC-OH-3'	5'-linker
**L5Bc**	5'-invddT-ACACrGrArCrGrCrUrCrUrUrCrCrGrArUrCrUrNrNrNrCrArCrUrArGrC-OH-3'	5'-linker
**L5Bd**	5'-invddT-ACACrGrArCrGrCrUrCrUrUrCrCrGrArUrCrUrNrNrNrUrCrUrCrUrArGrC-OH-3'	5'-linker
**L5Ca**	5'-invddT-ACACrGrArCrGrCrUrCrUrUrCrCrGrArUrCrUrNrNrNrCrUrArGrC-OH-3'	5'-linker
**L5Cb**	5'-invddT-ACACrGrArCrGrCrUrCrUrUrCrCrGrArUrCrUrNrNrNrUrGrGrArGrC-OH-3'	5'-linker
**L5Cc**	5'-invddT-ACACrGrArCrGrCrUrCrUrUrCrCrGrArUrCrUrNrNrNrArCrUrCrArGrC-OH-3'	5'-linker
**L5Cd**	5'-invddT-ACACrGrArCrGrCrUrCrUrUrCrCrGrArUrCrUrNrNrNrGrArCrUrUrArGrC-OH-3'	5'-linker
**L5Da**	5'-invddT-ACACrGrArCrGrCrUrCrUrUrCrCrGrArUrCrUrNrNrNrCrGrUrGrArUrN-OH-3'	5'-linker
**L5Db**	5'-invddT-ACACrGrArCrGrCrUrCrUrUrCrCrGrArUrCrUrNrNrNrGrCrArCrUrArN-OH-3'	5'-linker

**L5Dc**	5'-invddT-ACACrGrArCrGrCrUrCrU rUrCrCrGrArUrCrUrNrNrNrUrArGrUrGrC rN-OH-3'	5'-linker
**L5De**	5'-invddT-ACACrGrArCrGrCrUrCrU rUrCrCrGrArUrCrUrNrNrNrArUrCrArCrG rN-OH-3'	5'-linker
**L5Ea**	5'-invddT-ACACrGrArCrGrCrUrCrU rUrCrCrGrArUrCrUrNrNrNrCrArCrUrGrU rN-OH-3'	5'-linker
**L5Eb**	5'-invddT-ACACrGrArCrGrCrUrCrU rUrCrCrGrArUrCrUrNrNrNrGrUrGrArCrA rN-OH-3'	5'-linker
**L5Ec**	5'-invddT-ACACrGrArCrGrCrUrCrU rUrCrCrGrArUrCrUrNrNrNrUrGrUrCrArC rN-OH-3'	5'-linker
**L5Ed**	5'-invddT-ACACrGrArCrGrCrUrCrU rUrCrCrGrArUrCrUrNrNrNrArCrArGrUrG rN-OH-3'	5'-linker
**PRT_D01**	5′-CAAGCAGAAGACGGCATACGAGA TCGAGTAATCATTCCTGGCCTTGGCA CCCGAGAATTCCA-OH-3'	RT, indexing in same step
**PRT_D02**	5′-CAAGCAGAAGACGGCATACGAGA TTCTCCGGACATTCCTGGCCTTGGC ACCCGAGAATTCCA-OH-3'	RT, indexing in same step
**PRT_D03_RT**	5′-CAAGCAGAAGACGGCATACGAGA TAATGAGCGCATTCCTGGCCTTGGC ACCCGAGAATTCCA-OH-3'	RT, indexing in same step
**PRT_D04_RT**	5′-CAAGCAGAAGACGGCATACGAGA TGGAATCTCCATTCCTGGCCTTGGCA CCCGAGAATTCCA-OH-3'	RT, indexing in same step
**PRT_D05_RT**	5′-CAAGCAGAAGACGGCATACGAGA TAGCTTCAGCATTCCTGGCCTTGGCA CCCGAGAATTCCA-OH-3'	RT, indexing in same step
**PRT_D06_RT**	5′-CAAGCAGAAGACGGCATACGAGA TGCGCATTACATTCCTGGCCTTGGCA CCCGAGAATTCCA-OH-3'	RT, indexing in same step
**PRT_D07_RT**	5′-CAAGCAGAAGACGGCATACGAGA TCATAGCCGCATTCCTGGCCTTGGC ACCCGAGAATTCCA-OH-3'	RT, indexing in same step
**PRT_D08_RT**	5′-CAAGCAGAAGACGGCATACGAGA TTTCGCGGACATTCCTGGCCTTGGC ACCCGAGAATTCCA-OH-3'	RT, indexing in same step
**PRT_D09_RT**	5′-CAAGCAGAAGACGGCATACGAGA TTATTCAGGCATTCCTGGCCTTGGCA CCCGAGAATTCCA-OH-3'	RT, indexing in same step
**PRT_D10_RT**	5′-CAAGCAGAAGACGGCATACGAGA TATCGGTCCCATTCCTGGCCTTGGC ACCCGAGAATTCCA-OH-3'	RT, indexing in same step
**PRT_D11_RT**	5′-CAAGCAGAAGACGGCATACGAGA TGTAAATATCATTCCTGGCCTTGGCA CCCGAGAATTCCA-OH-3'	RT, indexing in same step
**PRT_D12_RT**	5′-CAAGCAGAAGACGGCATACGAGA TACGTTGACCATTCCTGGCCTTGGCA CCCGAGAATTCCA-OH-3'	RT, indexing in same step
**PPCR_r**	5′-CAAGCAGAAGACGGCATACGA-OH-3'	library PCR
**PPCR_f**	5′-AATGATACGGCGACCACCGAGAT CTACACTCTTTCCCTACACGACGCTC TTCCGATCT-OH-3′	library PCR
**Pi_index_read**	5′-GGAATTCTCGGGTGCCAAGGCCA GGAATG-OH-3'	custom index read primer
	**qPCR analyses**	
**Pq_CHO2_f**	5′-GGTTCTTCTGGTGTCCGTATTT-OH-3'	qPCR

**Pq_CHO2_r**	5′-GTGCCATCACCATACGTTCT-OH-3'	qPCR
**Pq_IRE1_f**	5′-CGGCAGATAGTGGAAGATGAAG-OH-3'	qPCR
**Pq_IRE1 r**	5′-TGGTTCTTGGAGCAGGAATG-OH-3'	qPCR
**Pq_RAT1_f**	5′-GACCACGCAAGGTACTTGTTA-OH-3'	qPCR
**Pq_RAT1_r**	5′-GCATCCCTAGCACTCCTAAATC-OH-3'	qPCR
**Pq_Ty_YLR035C-A_f**	5′-ACATAAGACCTCCACCACATTT-OH-3'	qPCR
**Pq_Ty_YLR035C-A_r**	5′-GTACCAGTTCGCTCCACTTT-OH-3'	qPCR
**Pq_HAC1_total_f**	5′-ACCAGGAAACTACAGTGAACAA-OH-3'	qPCR
**Pq_HAC1_total_r**	5′-CCATCAGAGAACCACGACTAAA-OH-3'	qPCR
**Pq_HAC1_ex_ex_f**	5′-CGATATAGCGGGAAACAGTCTAC-OH-3'	qPCR
**Pq_HAC1_ex_ex_r**	5′-TGACTGCGCTTCTGGATTAC-OH-3'	qPCR
**Pq_scR1_f**	5′-TCTGGCCGAGGAACAAATC-OH-3'	qPCR
**Pq_scR1_r**	5′-CGGTGCGGAATAGAGAACTATC-OH-3'	qPCR
**Pq_RPL19B_f**	5′-AACTAGAGCCCATGCTCAATC-OH-3'	qPCR
**Pq_RPL19B_r**	5′-CAGACGACTTGGGATGGTAAA-OH-3'	qPCR
	**Northern blots**	
**Np_GFP**	5′-ACCTTTTAACTCGATACGATTAACAAGGG-OH-3'	Northern probe
**Np_scR1**	5’-ATCCCGGCCGCCTCCATCAC-OH-3’	Northern probe
**Np_RPL19B**	5’-AGCTTCTCTAGTACCCTTTCTCT-OH-3’	Northern probe

SUPPLEMENTARY REFERENCES

1 Hartman, J. L. t. & Tippery, N. P. Systematic quantification of gene interactions by phenotypic array analysis. *Genome Biol* **5**, R49 (2004).
2 Duttler, S., Pechmann, S. & Frydman, J. Principles of cotranslational ubiquitination and quality control at the ribosome. *Mol Cell* **50**, 379-393 (2013).
3 Hoepfner, D. *et al.* High-resolution chemical dissection of a model eukaryote reveals targets, pathways and gene functions. *Microbiol Res* **169**, 107-120 (2014).
4 Brown, J. A. *et al.* Global analysis of gene function in yeast by quantitative phenotypic profiling. *Mol Syst Biol* **2**, 2006 0001 (2006).
5 Sitron, C. S., Park, J. H. & Brandman, O. Asc1, Hel2, and Slh1 couple translation arrest to nascent chain degradation. *RNA* **23**, 798-810 (2017).
