# Supplementary material for: Molecular interactions between Hel2 and RNA supporting ribosome-associated quality control
Source: Nat Commun. 2019 Feb 4;10:563. doi: 10.1038/s41467-019-08382-z (PMC6362110; doi:10.1038/s41467-019-08382-z)
Supplement: Supplementary file 3 — Reporting Summary [file 41467_2019_8382_MOESM3_ESM.pdf]

nature research

Corresponding author(s): David Tollervey

Reporting Summary

Nature Research wishes to improve the reproducibility of the work that we publish. This form provides structure for consistency and transparency in reporting. For further information on Nature Research policies, see Authors & Referees and the Editorial Policy Checklist.

Statistical parameters

When statistical analyses are reported, confirm that the following items are present in the relevant location (e.g. figure legend, table legend, main text, or Methods section).

n/a	Confirmed	
☐	☒	The exact sample size (*n*) for each experimental group/condition, given as a discrete number and unit of measurement
☐	☒	An indication of whether measurements were taken from distinct samples or whether the same sample was measured repeatedly
☐	☒	The statistical test(s) used AND whether they are one- or two-sided<br>*Only common tests should be described solely by name; describe more complex techniques in the Methods section.*
☒	☐	A description of all covariates tested
☒	☐	A description of any assumptions or corrections, such as tests of normality and adjustment for multiple comparisons
☐	☒	A full description of the statistics including central tendency (e.g. means) or other basic estimates (e.g. regression coefficient) AND variation (e.g. standard deviation) or associated estimates of uncertainty (e.g. confidence intervals)
☒	☐	For null hypothesis testing, the test statistic (e.g. *F*, *t*, *r*) with confidence intervals, effect sizes, degrees of freedom and *P* value noted<br>*Give P values as exact values whenever suitable.*
☒	☐	For Bayesian analysis, information on the choice of priors and Markov chain Monte Carlo settings
☒	☐	For hierarchical and complex designs, identification of the appropriate level for tests and full reporting of outcomes
☐	☒	Estimates of effect sizes (e.g. Cohen's *d*, Pearson's *r*), indicating how they were calculated
☐	☒	Clearly defined error bars<br>*State explicitly what error bars represent (e.g. SD, SE, CI)*

Our web collection on statistics for biologists may be useful.

Software and code

Policy information about availability of computer code

Data collection	Software used for data collection was the proprietory software that belongs to the machines described in the Methods section.
Data analysis	Most of the code used in this work is available online. Custom code can be made available upon request through the corresponding author.

For manuscripts utilizing custom algorithms or software that are central to the research but not yet described in published literature, software must be made available to editors/reviewers upon request. We strongly encourage code deposition in a community repository (e.g. GitHub). See the Nature Research guidelines for submitting code & software for further information.

Data

Policy information about availability of data

All manuscripts must include a data availability statement. This statement should provide the following information, where applicable:

- Accession codes, unique identifiers, or web links for publicly available datasets
- A list of figures that have associated raw data
- A description of any restrictions on data availability

The sequencing data have been deposited in NCBI's Gene Expression Omnibus and are accessible through GEO series accession number GSE114429.
The source data underlying Figs. 2a, 3a, 4a-b, 5, 6a-c, 7b-c, 8a-g, 9a-b and Supplementary Figs. 4, 6b, 7, 8, 9, 10a-c, 11a-c, 12a-l, 13a-i, 14, 15a-c, 16a-c are provided

as a Source Data file.

Field-specific reporting

Please select the best fit for your research. If you are not sure, read the appropriate sections before making your selection.

☒ Life sciences ☐ Behavioural & social sciences ☐ Ecological, evolutionary & environmental sciences

For a reference copy of the document with all sections, see nature.com/authors/policies/ReportingSummary-flat.pdf

Life sciences study design

All studies must disclose on these points even when the disclosure is negative.

Sample size	CRAC analyses were conducted at least in duplicate.<br>Growth tests were conducted at least in duplicate for strains that did not differ from wildtype. For strains that differed from wildtype, tests were conducted at least in triplicate, to improve statistical power.<br>Ribosome association assays, Western blot analyses of RQC and Northern blot analyses of NGD were performed in triplicate.
Data exclusions	In Northern blot analysis of NGD, average and standard deviation were calculated from the three replicates, except for non-stalling Hel2LKAA, where the third replicate was not taken into account due to low signal intensity.<br>In qPCR analyses, obvious outliers were removed from technical triplicates for average calculation from the biological replicates.
Replication	All findings reported were shown to be reproducible, as described in the manuscript.
Randomization	Randomization is not done in this type of yeast study.
Blinding	Blinding is not done in this type of yeast study.

Reporting for specific materials, systems and methods

Materials & experimental systems

n/a	Involved in the study
☒	☐ Unique biological materials
☐	☒ Antibodies
☒	☐ Eukaryotic cell lines
☒	☐ Palaeontology
☒	☐ Animals and other organisms
☒	☐ Human research participants

Methods

n/a	Involved in the study
☒	☐ ChIP-seq
☒	☐ Flow cytometry
☒	☐ MRI-based neuroimaging

Antibodies

Antibodies used	rabbit anti-TAP antibody (Thermo Scientific CAB1001); anti-Rabbit IgG (GE Healthcare NA934V); mouse anti-Pgk1 antibody (Thermo Fisher PA528612); anti-mouse IgG (GE Healthcare NXA931); peroxidase-anti-peroxidase antibody (Sigma Aldrich P1291); anti-GFP antibody (Roche, 11814460001)
Validation	TAP and GFP are species-independent.<br>anti-Pgk1 antibody species reactivity for yeast is confirmed on product homepage. According to manufacturer "Antibody specificity was demonstrated by siRNA mediated knockdown of target protein. HeLa cells were transfected with PGK1 siRNA and loss of signal was observed in Western Blot using Anti-PGK 1 Polyclonal Antibody". This antibody has been used routinely for yeast applications in our laboratory and the size of the band is consistent with Pgk1 size.
